# Synergistic effect of p-type and n-type dopants in semiconductors for efficient electrocatalytic water splitting†

**DOI:** 10.1039/d2sc04585k

**Published:** 2022-11-15

**Authors:** Tugce Kutlusoy, Spyridon Divanis, Rebecca Pittkowski, Riccardo Marina, Adrian M. Frandsen, Katerina Minhova-Macounova, Roman Nebel, Dongni Zhao, Stijn F. L. Mertens, Harry Hoster, Petr Krtil, Jan Rossmeisl

**Affiliations:** Center of High Entropy Alloy Catalysis, Department of Chemistry, University of Copenhagen Universitetsparken 5, København Ø 2100 Copenhagen Denmark jan.rossmeisl@chem.ku.dk; J. Heyrovsky Institute of Physical Chemistry, Academy of Sciences of the Czech Republic Dolejskova 3 Prague 18223 Czech Republic; New Application Research, Research and Development Division, Industrie De Nora S.p.A. 20134 Milan Italy; Department of Chemistry, Energy Lancaster and Materials Science Institute, Lancaster University Lancaster LA1 4YB UK; Fakultät für Ingenieurwissenschaften, Lehrstuhl Energietechnik, Universität Duisburg-Essen Lotharstra. 1 47048 Duisburg Germany

## Abstract

The main challenge for acidic water electrolysis is the lack of active and stable oxygen evolution catalysts based on abundant materials, which are globally scalable. Iridium oxide is the only material which is active and stable. However, Ir is extremely rare. While both active materials and stable materials exist, those that are active are usually not stable and *vice versa*. In this work, we present a new design strategy for activating stable materials originally deemed unsuitable due to a semiconducting nature and wide band gap energy. These stable semiconductors cannot change oxidation state under the relevant reaction conditions. Based on DFT calculations, we find that adding an n-type dopant facilitates oxygen binding on semiconductor surfaces. The binding is, however, strong and prevents further binding or desorption of oxygen. By combining both n-type and p-type dopants, the reactivity can be tuned so that oxygen can be adsorbed and desorbed under reaction conditions. The tuning results from the electrostatic interactions between the dopants as well as between the dopants and the binding site. This concept is experimentally verified on TiO_2_ by co-substituting with different pairs of n- and p-type dopants. Our findings suggest that the co-substitution approach can be used to activate stable materials, with no intrinsic oxygen evolution activity, to design new catalysts for acid water electrolysis.

## Abstract 2

Co-substituting a stable material, *e.g.* TiO_2_, with both n- and p-type dopants, allows tuning its reactivity to activate the material for oxygen evolution. This opens up a new design avenue for acid water electrolysis electrocatalysts.

## Graphical abstract

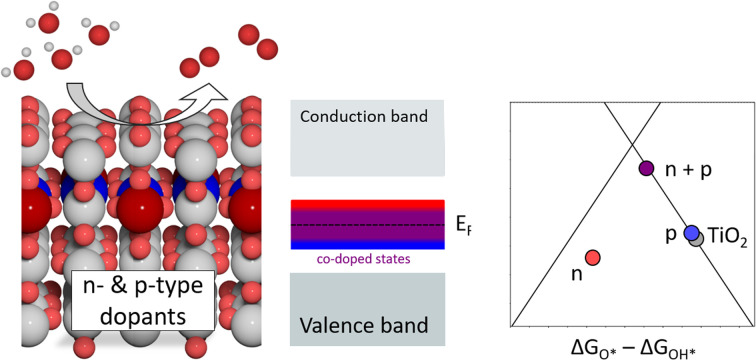

## Introduction

Large-scale exploration of renewable energy is vital for new sustainable energy concepts, meeting the requirement to remove the dependence on fossil fuels. Renewable energy, based on wind or solar, is affordable but suffers from their intrinsic intermittent nature and inconvenient regional distribution. These inherent disadvantages need to be mitigated by the design of novel energy storage concepts based on chemical bonds.^1^

Molecular hydrogen seems to be one of the primary candidates for a new energy storage system and energy vector.^2^ The only H_2_ production process fully compatible with carbon neutrality and sustainability requirements is water electrolysis, which converts electricity into chemical energy by splitting H_2_O into oxygen and hydrogen. Despite significant efforts devoted to its optimization, water electrolysis still needs to be fundamentally improved in terms of efficiency and durability.^1,2^

Water electrolysis can be performed both in alkaline as well as in acid environments.^3–7^ Regardless of pH, the efficiency of the overall process is controlled by the sluggish kinetics of the oxygen evolution reaction (OER) at the anode.^8^

Water electrolysis in acid media would in many cases be favored over that in alkaline media, because of more facile kinetics of the cathodic hydrogen evolution, high electrolyte conductivity, and high voltage efficiencies at high current densities.^9^ Despite being technologically promising, the use of acid water electrolyzers is limited by a lack of affordable, active, and sufficiently stable OER catalysts.^10^

The catalysts showing the best trade-off between durability and performance in oxygen evolution in acid media are oxides based on Ir or Ru.^7,11^ The low abundance and high price of these materials make the acid water electrolysis process practically unscalable.^12,13^

This fact stresses the importance of developing novel OER catalysts that are active, stable, and scalable for acid media oxygen evolution.^14^ The most straightforward improvement of feasibility can be achieved by an improvement of the stability while reducing or replacing the noble metal content in the catalysts.^15,16^

One can summarize the OER process as a sequence of four consecutive one-electron/proton transfer steps, which require the formation of three surface confined intermediates.^17,18^ The individual reaction steps are:

1 H_2_O + * → HO* + H^+^ + e^−^

2 HO* → O* + H^+^ + e^−^

3 H_2_O + O* → HOO* + H^+^ + e^−^

4 HOO* → * + O_2_ + H^+^ + e^−^

In an ideal catalyst all reaction intermediates, HO*, O*, and, HOO*, bind on the surface in such a way that all reaction steps (1)–(4) take place at the equilibrium potential of 1.23 V (*i.e.*, each reaction step is driven by the energy of 1.23 eV).

The main factor limiting the process optimization of water oxidation is the scaling relation between the first [HO*] and the third intermediate [HOO*].^19–21^ As both intermediates bind identically to the surface, a constant difference in adsorption energies of 3.2 eV is obtained, regardless of catalyst material. This situation greatly deviates from the ideal case where the difference in HO* and HOO* adsorption energies should equal 2.46 eV.^19,20^ The validity of the scaling relation has been shown also experimentally.^22,23^

To avoid the feasibility restrictions of the state-of-the-art OER catalysts based on Ir and Ru discussed above, we propose an alternative approach: activating semiconducting materials like, *e.g.*, TiO_2_, to form potent OER catalysts of high stability. The stability of many semiconducting oxides is inherently related to their wide bandgap, which, in turn, leads to low catalytic activity. The poor performance of TiO_2_, chosen as a model semiconductor, in electrochemical water oxidation is well established.^24–29^ Doping TiO_2_ to increase its OER activity is also a well-established technique.^28–33^

This work demonstrates the activation of TiO_2_ for OER catalysis by subsituting Ti-atoms with both n- and p-type dopants.^34–36^ Such co-substitution has never been – to the best of our knowledge – applied in electrocatalysis. Here, we present a theoretical approach outlining a systematic way to improve the catalytic activity of semiconductors through co-substitution. The co-substitution approach optimizes the trade-off between stability and activity by preserving the intrinsic stability of the semiconducting material while increasing its activity because of the electronic states created by the dopants. The theoretical predictions are experimentally verified on model catalysts based on co-substituted TiO_2_.

## Results and discussion

### Co-substitution approach

For semiconducting oxides, any computational approach needs to avoid finite-size effects leading to computational artefacts^20^. With the elimination of the finite size effect, it is possible to computationally assess activity trends of heavily substituted semiconductors, *e.g.* TiO_2_, and position these materials on the theoretical OER activity volcano to draw a comparison with other materials. The mechanism of TiO_2_ activation *via* co-substitution is shown on the rutile polymorph to maintain structural similarity with the state-of-the-art OER catalysts in acid media (RuO_2_, IrO_2_).

**Fig. 1:**
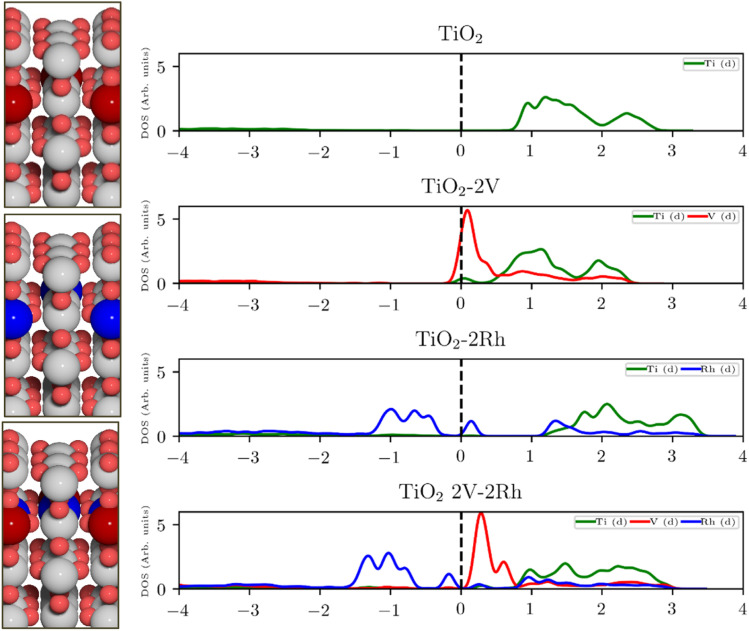
Projected density of states (pDOS) of pristine and substituted TiO_2_ and corresponding atomic structures of rutile-type TiO_2_ (110) with V (red), Rh (blue) (from top to bottom: n-substituted, p-substituted, co-substituted). pDOS from top to bottom: pristine TiO_2_; V-substituted TiO_2_: the V d-states intersect with the Fermi level. The majority of the states is located at energies above the Fermi level; Rh-substituted TiO_2_: the Rh d-states are located mostly around the Fermi level and also at higher energies above the Fermi level; and V–Rh co-substituted TiO_2_. The dopants' d-states populate the bandgap and are placed around the Fermi level.

The poor conductivity of TiO_2_ can be improved by n-type substitution (*i.e.*, by adding donors of electrons), which can directly provide electrons to the conduction band (CB) or create states in the bandgap close to the CB edge. The effect of the n-type dopants can be demonstrated on a model system, where TiO_2_ is substituted with V. Extending the analysis to p-type substituted TiO_2_ (*i.e.*, introducing acceptors of electrons *e.g.*, Rh) one finds a different type of behavior. p-Type dopants increase the probability of forming a hole in the valence band (VB). In other words, p-type dopants deplete the electron density of the VB forcing the Fermi level to move close to the electronic states created by the p-type dopants. The effect of substitution on the TiO_2_ electronic structure is schematically depicted in Fig. 1, which shows the projected density of states (pDOS) of the TiO_2_ rutile structure substituted with n-type (V) and p-type dopant (Rh), as well as the co-substituted structure (V + Rh). Both substituting and co-substituting the TiO_2_ with Rh and V create states in the bandgap, which due to their occupancy may interact with the reactant water molecules. These newly created states may eventually (in the case of co-substitution) form a new, rather narrow, electronic band that crosses the Fermi level. In this way, the electronic structure of the co-substituted TiO_2_ resembles that of a conductor. The behavior of the Fermi level, which varies according to the type of dopant introduced, is following literature.^37^

**Fig. 2:**
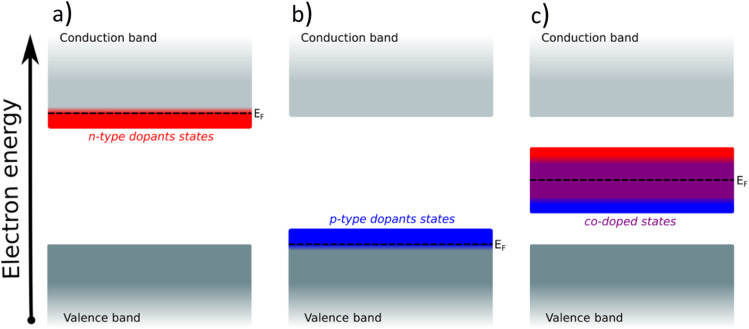
(a) Insertion of an n-type dopant in a semiconductor, (b) insertion of a p-type dopant in a semiconductor, (c) simultaneous insertion of an n-type and a p-type dopant in a semiconductor and the creation of the compensated electron states.

Creating these pseudo-conductor states in the bandgap of a stable, essentially semiconducting, material such as TiO_2_ has a fundamental impact on the catalytic behavior of these materials. The substitution/co-substitution approach allows, along with conductivity optimization, tuning of the surface reactivity. Most n-type dopants can easily provide an electron to the binding site. Combining n- and p-type dopants leads to a situation when it becomes increasingly difficult for electrons to leave the dopant complex and participate in the surface binding. Therefore, the co-substitution strategy may be able to make stable semiconducting oxides active for OER catalysis (see Fig. 2).

**Fig. 3:**
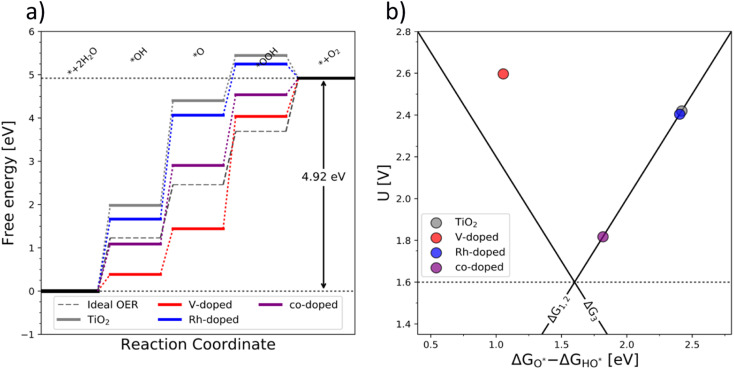
(a) Free energy diagram of the pure (grey), single substituted (blue – Rh and red – V), and co-substituted (purple) TiO_2_ and (b) the corresponding activity volcano depicting the increased catalytic performance of the co-substituted structure.

The OER free energy diagrams presented in Fig. 3a identify the oxidation of HO* to O* as the potential determining step (PDS) for pristine TiO_2_ (eqn (2)). This places the rutile polymorph of TiO_2_ on the weak binding branch of the OER activity volcano (see Fig. 3b, grey). The n-type dopants change the PDS from the HO* → O* oxidation to O* → HOO*. This shifts the n-substituted TiO_2_ from the weak binding branch to the strong binding branch of the activity volcano (Fig. 3b, red data point). Hence, n-type substitution makes the rutile structure highly reactive resulting in too strong oxygen adsorption. This causes an accumulation of chemisorbed oxygen intermediates eventually blocking the surface. After blocking the surface, the activity of the n-type substituted rutile does not differ from that of pristine TiO_2_ (see Fig. 3b).

DFT analysis of the effect of p-type dopants on the theoretically assessed OER activity places the p-substituted TiO_2_ on the weak binding side of the volcano and the predicted activity shows only a marginal improvement over that of pristine TiO_2_. Therefore, in conclusion, the substitution of TiO_2_ with a single type of dopant does not improve its OER activity since (i) the n-type substitution causes primarily an accumulation of strongly chemisorbed oxygen on the surface and (ii) the activity of the p-type substituted materials does not significantly change the activity of the original material.

After examining the effect of both p-type and n-type substitution on the OER activity of the TiO_2_ surface, one may naturally consider simultaneous co-substitution of n- and p-type elements. The OER-related catalytic activity of the co-substituted TiO_2_ is represented in Fig. 3 by the purple-colored data. The theoretical activity of co-substituted TiO_2_ significantly differs from those of pristine as well as n- and p-type substituted TiO_2_. The theoretical prediction places the co-substituted TiO_2_ on the weak branch of the volcano; however, the theoretical overpotential is greatly reduced, mainly due to an optimized binding of the O* intermediate (Fig. 3a).

### Coulombic effect

The improved binding of OER intermediates at the co-substituted TiO_2_ surface retains a local character that is dependent on the relative position of the dopants in the structure. To investigate this effect, we performed a series of calculations for different local arrangements of both dopants in the unit cell by varying the relative distance of the dopants as well as their distance to the binding site. Based on the relative position of both Rh and V, the resulting catalysts may reside on either branch of the theoretical volcano (see ESI, Fig. S1†).

**Fig. 4:**
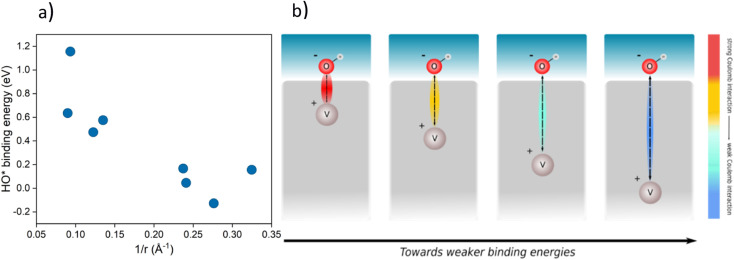
(a) Binding energy of HO* against 1/*r*, where *r* is the distance of the dopant (V) from the intermediate in a TiO_2_ structure for various placements in the structure (cus and bridge sites, the corresponding structures are given in ESI, Fig. S2†) (b) schematic representation of the effect of the distance between the intermediate (HO*) and the dopant (V) on the binding energy of the intermediate.

A phenomenological explanation of this effect is the varying electrostatic interaction which changes with the distance of the dopant to the adsorbate. To further investigate this phenomenon, we conducted calculations with a single V atom in the TiO_2_ structure, when the distance between the dopant and the adsorbed intermediate was gradually increased. We selected HO* as the model intermediate since it is bound on the surface *via* a single bond. In this way, the use of a single dopant does not introduce finite-size effects. The corresponding atomic structure representations are given in the ESI, Fig. S2.† By varying the distance of dopants to the adsorbed intermediate, the binding energy of the intermediate changes, which has previously been shown for TiO_2_ substituted with molybdenum.^38^ In particular, we find that a linear correlation exists between the HO* binding energy and the reciprocal distance between the dopant and the binding site. The HO* binding energy shows the same variation with the reciprocal distance of the vanadium atom and binding titanium atom, as if V delivers an electron to HO*, see Fig. 4a. The generality of the coulombic effect between the dopant and the binding intermediate can be shown by extending the analysis to SrTiO_3_, see ESI Fig. S3,† where the same type of dependence of the HO* binding energy on the reciprocal distance 1/*r* is obtained. The interaction between V^+^ and HO^−^ is thus a simple coulombic term; the closer the dopant is to the binding site the stronger the binding due to the attraction between the negative and positive charges (see Fig. 4b).

Extending this approach to the co-substituted model, one finds the reactivity of the binding site to depend on the distance to both dopants, the relative distance of dopants, and the dielectric constant of the semiconductor. It is more complicated to show the coulombic interaction directly for the co-substituted case, as it will depend on the partial charge and the dopants' and adsorbate positions, respectively. The complexity of the model is likely to increase with an increasing number of dopants. This does not neglect the role of the nature of the dopant in binding energy control, as this determines the position of the states in the bandgap. It means, however, that for a given set of dopants, semiconductors, and intermediates the variations in binding energies are governed by electrostatics. A more detailed discussion of the coulombic effect further showing the generality of the concept irrespective of the dopant is given in the ESI, see Fig. S1–S10† for both substituted and co-substituted TiO_2_.

**Fig. 5:**
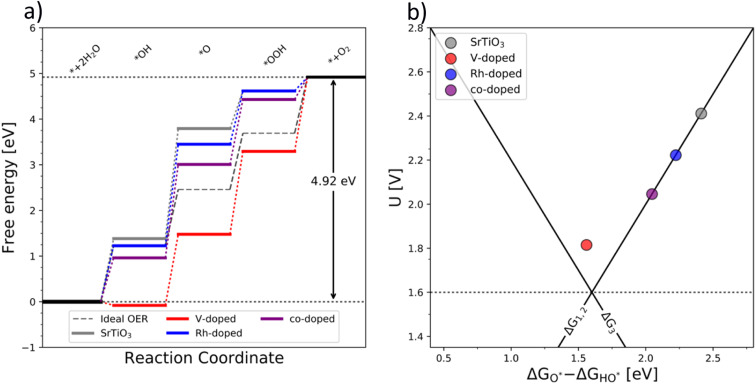
(a) Free energy diagram of the pure (grey), substituted (blue and red), and co-substituted (purple) SrTiO_3_. As in the case of TiO_2_, the energy level of the intermediates corresponding to the co-substituted structure are in between the p- and n-states (b) the corresponding activity volcano.

The generality of the co-substitution concept can be further demonstrated by extending it to another semiconducting system of significant stability – SrTiO_3_ perovskite. The chosen perovskite structure shows (in its non-substituted state) a band structure similar to that of TiO_2_. It also shows outstanding chemical stability but its OER-related electrocatalytic activity is rather low, in part also due to its wide bandgap.^39,40^ As in the case of TiO_2_, Rh and V were used as the p-type and n-type dopant, respectively. The results of the thermodynamic analysis of the OER catalysis on pristine and substituted SrTiO_3_ structures are summarized in Fig. 5.

**Fig. 6:**
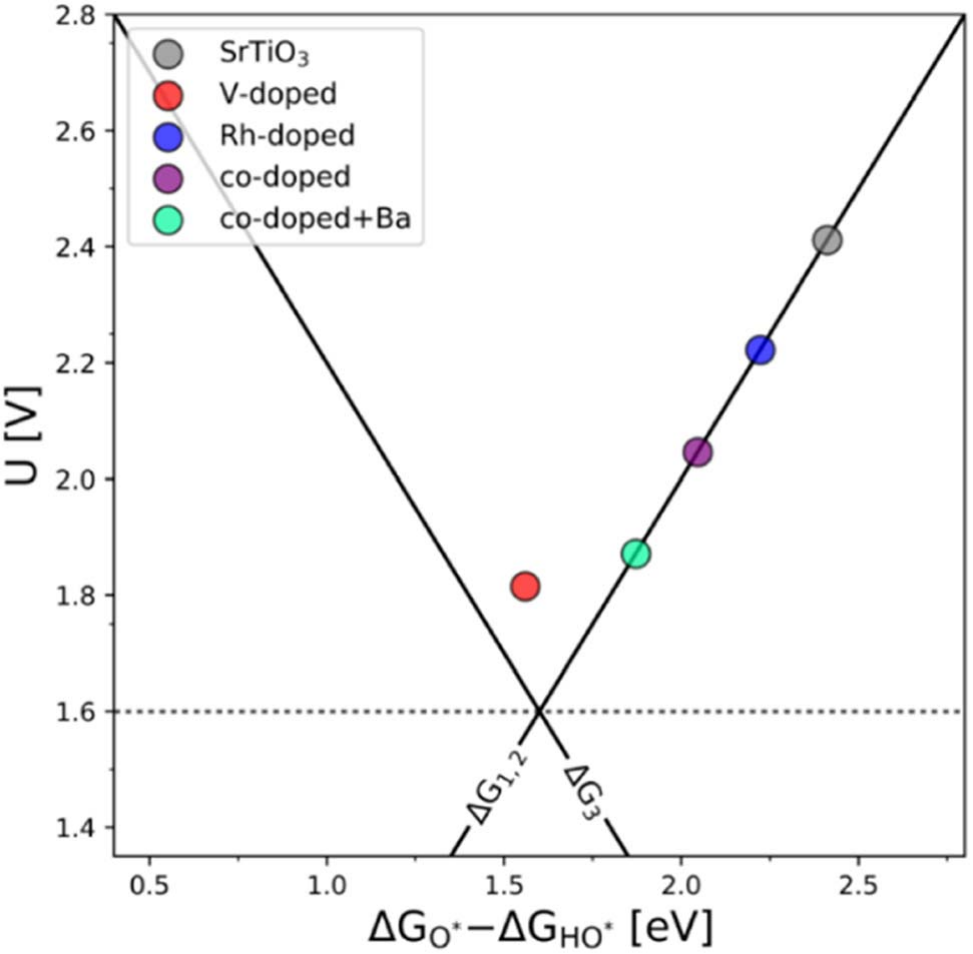
The OER activity volcano of V-substituted, Rh-substituted, and V–Rh co-substituted SrTiO_3_ with the addition of the V–Rh co-substituted Sr_0.84_Ba_0.16_TiO_3_ structure, which in terms of activity it resembles the one of the co-substituted TiO_2_.

The results presented in Fig. 5 essentially reproduce the trends predicted for the substituted and co-substituted TiO_2_. The only difference in the behavior of SrTiO_3_ is that n-type substitution alone improves the OER activity more than the co-substituted SrTiO_3_. This observation shows that the partially oxidized SrTiO_3_ corresponds to the most stable phase in the overall OER reaction sequence. The co-substituted structure lies energetically in between the p- and the n-type substituted SrTiO_3_ as in the case of TiO_2_ (Fig. 5). The theoretical activity of the co-substituted SrTiO_3_ is much lower than that of co-substituted TiO_2_. This unfavorable trend can, however, be reverted by partial substitution of Sr with Ba in the A-site of the perovskite structure, which activates the co-substituted perovskite in the OER process (Fig. 6). Schematic representation of the co-substituted perovskite structures SrTiO_3_ and Ba_0.16_Sr_0.84_TiO_3_ are shown in the ESI, Fig. S11.†

An improvement of the OER activity of perovskite-based catalysts connected with control of the redox state of the B-site cation in the perovskite structure has previously been reported.^3^ This, however, does not explain the observed effect, since the Ba s-valence orbitals are not catalytically active. A possible explanation could be that alteration in the overall band structure occurs due to the Ba s-orbitals pushing the electronic states of both dopants to a more favorable position to facilitate the adsorption of OER intermediates.

To further test the generality of the co-substitution concept, we have broadened the initial screening of n- and p-type dopants of TiO_2_ and SrTiO_3_ to include V, Nb, Ta, Mo, and W, as n-type dopants, and Rh, Mn, Pd, and Ru as p-type dopants. Extending the co-substitution hypothesis, we have chosen the following additional pairs of p and n-type dopants: all n-type dopants (V, Nb, Ta, Mo, W) are paired with Rh, Mn, Pd, Ru. All combinations of p- and n-type dopants lead to electronic structures, which in their OER activity surpass the OER activity of pristine TiO_2_. (See Fig. S12 in the ESI†).

Importantly, the calculated bandgap energies of TiO_2_ and SrTiO_3_ are significantly underestimated compared to experimental values, which is a well-known problem of DFT.^41^ This may directly affect the calculated binding energies of the OER intermediates. *U*-correction partially compensates for the deviation of calculations from experiments,^37,42^ but this approach also has its drawbacks. The *U*-value used for each dopant is different, thus comparing the activity of different systems is not straightforward. In addition, the binding energy of the intermediates scales with the value of *U*-correction. The details of *U*-corrected bandgap energies are given in the ESI, Fig. S13–S15.† The absolute binding energies may be affected by systematic errors in the simulations and the inherent uncertainty of the position of individual combinations of n- and p-type dopants. However, the effect of the co-substitution will apply even if the absolute values change. Hence, the computational screening approach presented here allows for narrowing the selection of prospective dopants.

**Fig. 7:**
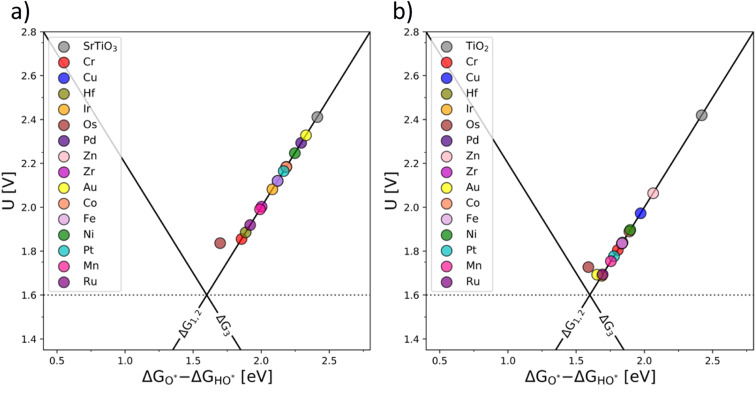
OER activity volcanoes for Sr_0.16_Ba_0.84_Ti(RhV)O_3_ (a) and Ti(RhV)O_2_ (b) substituted with extra transition metal dopants (additional d-states).

An ultimate step in the co-substitution optimization of OER activity of semiconductors can be seen in further substitution of the already co-substituted structures Ti(RhV)O_2_ and Ba_0.16_Sr_0.84_Ti(RhV)O_3_, with both n-type and p-type dopants. The results of this computational approach are summarized in Fig. 7, showing the position of these materials on the theoretical volcano. For both rutile and perovskite structures, introducing several extra dopants can further increase the OER activity of the co-substituted structures. This can be traced back to variations in the band structure as a result of adding the extra dopant. The Rh and V co-substitution states dominate however the behavior, which places all structures on the weak binding side of the OER volcano.

It may be concluded that the activity improvement reflected in Fig. 7 results from the joint effect of the compensated states and the additional d-states provided by the extra dopant to the active part of the system's band structure. A representative example of the pDOS for such a system is shown in the ESI, Fig. S16,† for the case of Mn, V, and Fe dopants introduced in the TiO_2_ structure.

While the theoretical analysis suggests that co-substituted TiO_2_ has the potential to disrupt the OER catalyst design, the viability of the concept needs to be experimentally verified. While the theoretical screening of prospective co-substituted systems is relatively affordable, the actual synthesis of the theoretically predicted systems is less straightforward. To prove the theoretical concept, a system is required that reflects the main features of the theoretical concept yet maintains a sufficient synthetic facility. Also, the theoretically conceived co-substituted materials represent metastable phases and, thus, the experimental proof of concept must rely on a low-temperature synthetic approach,^43,44^ favoring TiO_2_-based systems over SrTiO_3_-based materials.^45^

Experimental confirmation of the predicted superior OER activity of the co-substituted TiO_2_ materials is still relatively complicated. It needs to be noted that rutile, which is the basis of the theoretical analysis (it represents the thermodynamically stable titania polymorph), is difficult to prepare at low temperatures. The absence of convenient low-temperature synthesis also complicates the possible stabilization of large amounts of substituting cations in its structure, since the theoretically predicted chemical compositions are likely to remain stable only if the thermal treatment does not exceed 500 °C. These synthetic approaches, however, yield the thermodynamically metastable anatase titania polymorph.^45,46^ This fact should not present a fundamental problem since anatase is also an n-type semiconductor and features a similar bandgap and similar band edge energies as the rutile polymorph.^47,48^ To confirm that the co-substitution approach is transferable from the rutile model system and can be extended to the anatase polymorph, the adsorption characteristics of co-substituted anatase structures were computed (see ESI, Fig. S17†). It is shown that the same trends are observed for both major titania polymorphs (rutile and anatase) for both band structures and OER activity trends. Hence, the co-substitution approach can be generalized.

**Fig. 8:**
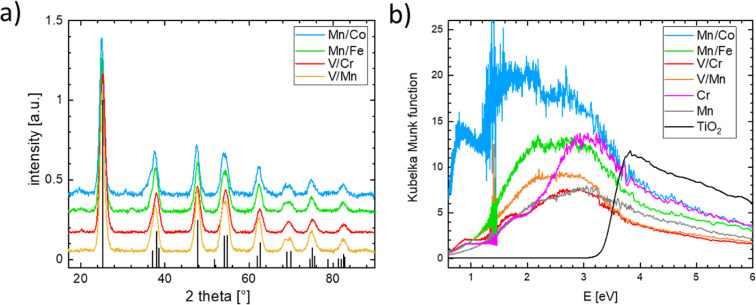
(a) X-ray diffraction patterns of the synthesized n and p co-doped TiO_2_ nanoparticles, the reference pattern for TiO_2_ in anatase structure is included as the black line pattern and (b) UV-vis spectra of all synthesized doped TiO_2_ nanoparticles including the pristine TiO_2_ anatase reference.

Single-substituted and co-substituted TiO_2_ nanoparticles were prepared using the spray-freeze freeze-dry approach.^49,50^ The substitution levels achieved in all prepared TiO_2_ materials were equal to a substitution of 20% of cationic positions, either with one dopant (Cr or Mn) or two dopants (both n- and p-type substitution) in equal amounts. All synthesized titanium oxide materials were nanocrystalline and conform to the anatase structure (see Fig. 8a). Substitution of an element in the anatase structure did not cause any significant variation in the lattice parameters, the characteristic particle size, and the coherent domain sizes. Details of the structural characterization of the obtained materials are given in the ESI (see Fig. S18–S21 and Table S1).† Although the XRD-based structure of the prepared materials conforms to that of the anatase TiO_2_ polymorph, the same cannot be said of their electronic structure as can be inferred from measured UV-vis spectra (see Fig. 8b). The UV-vis spectrum of pure anatase is typical for a wide bandgap semiconductor characterized by a clearly defined band gap of more than 3 eV. This type of behavior is not replicated in the substituted or co-substituted materials. The pronounced onset of the absorption in the visible region is already evident in the materials substituted with a single element (Mn, Cr in Fig. 8b). For materials subject to simultaneous n- and p-type substitution, one observes a continuous absorption extending over a rather broad interval of energies, spanning from circa 2.0 to 2.5 eV. This broad absorption band is further complemented by a resolved localized absorption band located at *ca.* 1 eV, characteristic namely for materials substituted with Co. This type of behavior is generally compatible with the conductivity behavior predicted in the DFT calculations. It needs to be noted that the co-substituted TiO_2_ materials do not retain the behavior characteristic of the pure TiO_2_, namely, they do not show characteristic photo-electrochemical activity.

**Fig. 9:**
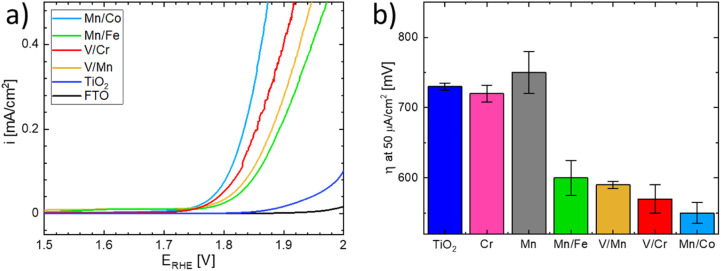
(a) Linear sweep voltammograms of the synthesized co-substituted TiO_2_ nanoparticles and the TiO_2_ benchmark material. Voltammograms were recorded in 0.1 M HClO_4_ with a scan rate of 5 mV s^−1^ using FTO as a current collector and (b) overpotential *η* necessary to drive a current density of 50 μA cm^−2^ on the co-substituted TiO_2_ nanoparticles, single dopant TiO_2_ nanoparticles, and the TiO_2_ benchmark sample. The values included are the mean averages of three individual measurements. The assignment of individual samples is given in the figure legend.

The OER activity of the substituted TiO_2_ materials was assessed in acid media and compared with that of the benchmark TiO_2_ sample (see Fig. 9).^51^ Details of the electrochemical characterization are given in the Experimental section. The linear sweep voltammograms of the co-substituted TiO_2_ samples are presented in Fig. 9a. For better comparison, the potential necessary to drive a current density of 50 μA cm^−2^ is taken as an estimate of the oxygen evolution activity (Fig. 9b). The estimated overpotentials show that the behavior of materials substituted with a single cation and that of the n- and p- co-substituted materials significantly differs, despite the similarity in UV-vis spectra. While the co-substituted materials clearly outperform the pure TiO_2_ benchmark sample, the activity of the materials substituted with a single cation remains comparable with that of the benchmark, *i.e.*, non-substituted TiO_2_. It can be concluded that only co-substitution has a pronounced effect on the OER activity of the TiO_2_ electrodes (see Fig. 9b). Although the observed behavior is essentially in line with the DFT-based theoretical prediction presented above, the actual OER activity seems to also be affected by the chemical nature of the dopants.

The Mn/Co co-substituted structure apparently exhibits the highest activity (*i.e.*, the lowest overpotential) among all the co-substituted structures as shown in Fig. 9b. On the other hand, the large variability of the activity observed in different experiments suggests that the synthesized materials may change their activity under operando conditions in acid media. This could particularly be the case for materials containing Mn and Co since oxides based on these elements are relatively good catalysts for OER,^52–56^ hence the observed enhanced activity may be attributed to a local level phase separation. Such a phase separation could, however, also cause material instability when the observed anodic current might be attributed to Mn or Co oxide leaching rather than to actual water oxidation. On the other hand, the second-best performance is exhibited by the V/Cr co-substituted structure, and since the oxides of V and Cr do not form good OER catalysts in themselves,^19,57,58^ one can argue that the compensation effect is responsible for their increased catalytic activity.

**Fig. 10:**
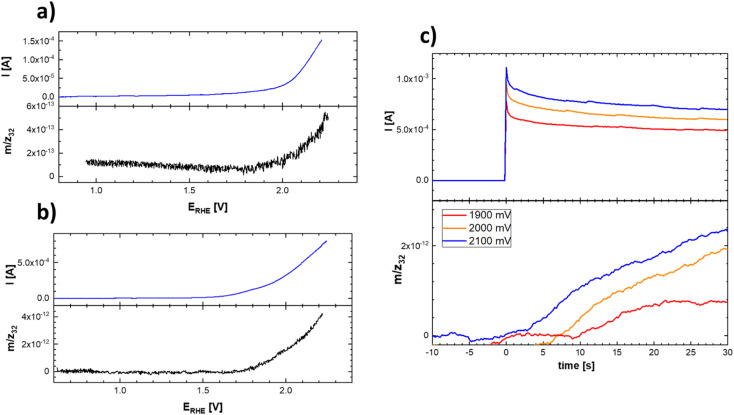
Linear scan voltammograms (top) and corresponding potential course of the mass spectrometric signal (bottom) of the evolved oxygen on Ti–Co–Mn (a) and Ti–Mn–V (b) in 0.1 M HClO_4_. Experimental conditions were identical as those indicated in Fig. 9 and (c) chronoamperometric curves and corresponding time course reflecting the oxygen evolution on Ti–Mn–V based catalyst in 0.1 M HClO_4_ at different potentials. The actual potentials (*vs.* RHE) are indicated in the figure legend.

This uncertainty can be resolved by separately following the actual oxygen formation during anodic polarization of the prepared materials and the chemical stability of the catalysts during oxygen evolution. The oxygen formation can be followed using online mass spectrometric detection by Differential Electrochemical Mass Spectrometry (DEMS), as depicted in Fig. 10.

**Fig. 11:**
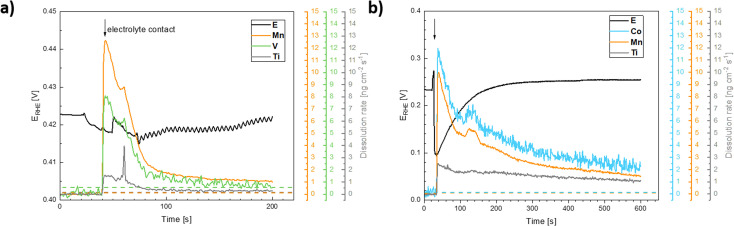
Evolution of the electrode potential (*E* (V) *vs.* RHE, left axis) and ICP-OES signals of the dissolution of the catalysts components following exposure of (a) the Ti–Mn–V and (b) the Ti–Mn–Co to 0.05 M H_2_SO_4_ electrolyte solution. The ICP-OES signals were converted to dissolution rates.

**Fig. 12:**
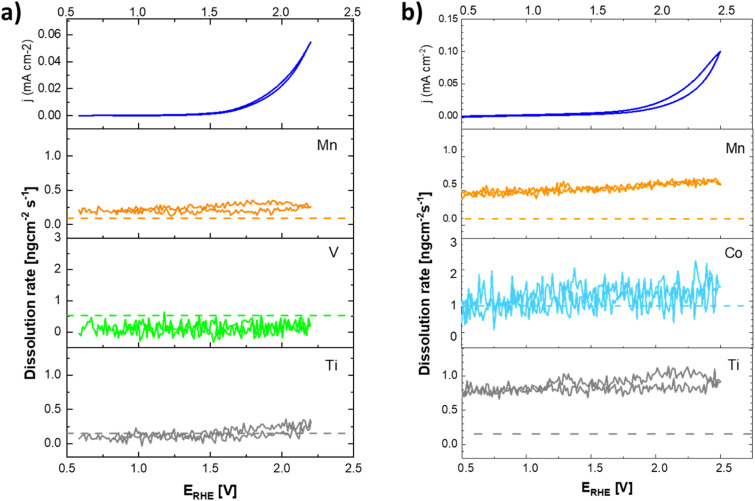
Cyclic voltammogram (top) and ICP-OES signals of the dissolution of the catalysts components during cyclic polarization of (a) the Ti–Mn–V and (b) the Ti–Mn–Co catalyst in 0.05 M H_2_SO_4_. Dashed lines indicate the ICP-OES detection limits.

The data presented in Fig. 10 conclusively show that the anodic current observed at potentials positive to 1.8 V *vs.* RHE is connected with the formation of oxygen. The formation of oxygen varies on fresh electrodes but becomes fairly reproducible during repetitive polarization experiments. Although the signal of the produced oxygen generally tracks the observed anodic current (see Fig. 10c), it cannot be used to prove if the oxygen evolution proceeds quantitatively. Anodic dissolution of the electrode materials cannot be deduced from DEMS data, so the possibility of parasitic catalyst corrosion requires a separate online ICP-OES experiment, summarized in Fig. 11 and 12.

Based on these data, the biggest change in the composition of the electrode is triggered by the first contact of the electrode materials with the electrolyte solution, which results in an enrichment of the electrolyte solution with all metal components of the prepared catalyst. A comparison of the behavior of two chosen representatives of co-substituted materials Ti–Mn–Co and Ti–Mn–V phases, however, clearly outlines a qualitatively different stability behavior of the Co and Mn-containing phases. Although the dissolution rate upon contact with sulphuric acid-based electrolyte is essentially the same for both systems, dissolution over time is more pronounced for the system containing Co than for that featuring V. The leaching of the catalysts' components into electrolyte solution starting upon contact is practically finished within the first 200 seconds in the case of material containing V, while the solution enrichment with metal cations in the case of the material containing Co still proceeds at an appreciable rate after 700 s. Regardless of the overall chemical composition, the ICP-OES data clearly shows that even Ti, generally considered to be stable in acid media, is not stable in the prepared catalysts. Its dissolution rate, however, remains about 3–5 times lower than that of the other catalyst components – Mn and Co. The contact of the catalyst with the electrolyte solution triggers a temporary potential fluctuation (rather small in the case of the Ti–Mn–V system) but the potential stabilises within *ca.* 5 minutes. It needs to be noted that the leaching of the Mn and Co is more pronounced than that of V.

The anodic polarization of the catalyst to potentials relevant to oxygen evolution reaction triggers additional changes in the chemical composition of the sample (see Fig. 12). In the case of catalysts in the Ti–Mn–V system, the anodic polarization in 0.05 M sulphuric acid does not lead to any appreciable transition metal leaching at potentials below 1.8 V *vs.* RHE (see Fig. 12).

At potentials above 1.8 V, Ti and Mn levels in solution increase above the detection limit. No leaching of V is, however, observed under these conditions. Interestingly, the rate of dissolution of the unstable component of the catalyst (Mn) is proportional to that of the stable Ti. Further, the dissolution rate of the Ti and Mn is not a simple function of the electrode potential, as a notable hysteresis between the anodic and cathodic scans of the voltammogram occurs. The microscopic state of the surface and how this state is approached therefore plays a role, similar as was observed during charging/discharging of LiMnO_2_.^59^

In the case of catalysts in the system Ti–Mn–Co (see Fig. 12b) we observed generally the same trends, as the anodic polarization to the potential of oxygen evolution does not trigger any appreciable additional catalysts dissolution. A constant, minor dissolution of Mn, Ti, as well as Co is however observed. This dissolution signal is, however, significantly smaller than the initial dissolution of the material upon contact with the electrolyte.

The formation of dissolved Ti-containing species is somewhat surprising given the expected stability of Ti oxides in acid media. The observed behavior, when the same rate of dissolution is observed for the reactive transition metals (Mn, Co) as for the apparently stable Ti, suggests that the conditions of the oxygen evolution process promote a formation of Ti peroxo-complexes, which are known to be fairly soluble under the conditions of the experiment.^60^ The destabilization through formation of Ti peroxo-complexes confirms that through the co-substitution approach Ti is indeed activated as a binding site for the oxygen intermediates in the catalytic cycle of the oxygen evolution process.

In summary, our data show that the anodic current recorded during the catalyst testing originates from the oxygen evolution process. Although the prepared materials are intrinsically unstable as shown by ICP-OES under OCP conditions, the data obtained at anodic polarization suggest that the materials stabilize themselves rather fast after exposure to the electrolyte solution, which may point to passivating behavior.^59^ The observed instability of the prepared substituted and co-substituted TiO_2_ nanoparticles can be explained by the random distribution of dopants in the particles. Dopants placed on the particle surface lead to leaching and loss of the dopants which in turn decreases the OER activity. Choosing a synthetic strategy that confines the dopants to the bulk^39^ as an alternative could be an approach to increasing the stability of these materials.

## Computational methods

The grid-based projector augmented wave (GPAW)^61,62^ package along with the atomic simulation environment (ASE)^63^ interface, was used for the density functional theory (DFT) calculations. The exchange and correlation energy of the electrons was expressed within the concept of the generalized gradient approximation (GGA) by implementing the RPBE^64^ functional. The grid spacing was selected to be *h* = 20 Å while the atomic positions were relaxed until the total forces were lower than 0.05 eV Å^−1^. A 4 × 4 × 1 Monkhorst–Pack^65^*k*-point sampling for the 1 × 3 replicated TiO_2_ rutile (110) surface was used, with the last two layers (out of four) of the super cell being constrained. The cubic configuration of the perovskite unit cell was used as the initial structure for the SrTiO_3_. A 10 × 10 × 10 *k*-point mesh with a plane wave energy cut-off of 900 eV and the RPBE functional were used to optimize the bulk structures. For optimizing the (100) SrTiO_3_ surface the same parameters as in the case of rutile were used for a direct comparison between the two structures.

## Experimental details

All materials were prepared by spray-freeze freeze-dry synthesis. A total metal concentration of 8 mM was maintained in all experiments. Titanium(iv) bis(ammonium lactato)dihydroxide (TBALD) solution (50 wt% in H_2_O, Sigma Aldrich) was used as the source of titanium. 40 mM concentrated stock solutions of TBALD in Milli-Q quality deionized water were prepared. As the source of transition metal dopants, vanadium(v) oxide (>98%), chromium(iii) nitrate nonahydrate (99%), manganese(ii) acetate tetrahydrate (>99%), cobalt(ii) acetate tetrahydrate (>98%), and iron(ii) acetate (>95%) were all purchased from Sigma Aldrich and used as received. The precursor solutions for each experiment were prepared by dissolving the corresponding metal salts in Milli-Q quality deionized water and adding the respective amount of TBALD stock solution as the titanium source. The ratio of the initial metal precursors was adjusted to achieve the desired nominal composition; a total amount of 20 at% dopants to titanium. For co-substitution experiments, the dopants were added in a 1 : 1 ratio. The solutions were stirred for 30 minutes before the spray-freezing step. The ice precursors were prepared by spraying 100 mL of precursor solution into *ca.* 2 L of liquid nitrogen. The obtained ice precursor was freeze-dried using a FreeZone Triad Freeze Dry System 7400030 (Labconco) at reduced pressure (1 Pa) according to the following temperature protocol: the temperature was kept constant at −30 °C while the cooling chamber was evacuated, followed by a gradual increase of the temperature [−30 °C (2 h), −25 °C (5 h), −20 °C (4 h), −15 °C (6 h), +30 °C (4 h)]. The obtained foam-like precursor was carefully removed from the freeze-dryer and annealed at 500 °C for 2 h in a muffle furnace.

The crystallinity and phase purity of the synthesized materials were analyzed by powder X-ray diffraction (XRD). The diffraction patterns were recorded using a Rigaku Miniflex 600 powder X-ray diffractometer with Cu K_α_ radiation operating at 30 kV and 10 mA. Le Bail fits were performed to determine the unit cell parameter with the Profex 3.13.0 software package^66^ based on the BGMN program.^67^

The morphology and particle size of all prepared samples were analyzed by scanning electron microscopy (SEM) using a Hitachi S4800 scanning electron microscope equipped with a Nanotrace EDX detector (Thermo Electron). The average sample composition was determined by energy-dispersive X-ray spectroscopy (EDX) measured at an accelerating voltage of 25 keV. The bandgap of the synthesized samples was determined by UV-vis diffuse reflectance spectroscopy (DRS) (PerkinElmer LAMBDA 950).

The oxygen evolution activity of the prepared materials was assessed for all materials. The TiO_2_ electrodes were prepared by drop-casting on fluorine-doped tin oxide (FTO) glass (TEC 15, Dyesol, 15 Ω per sq). The catalysts suspensions were prepared by dispersing 10 mg of catalyst in a solution of 1 mL H_2_O, 4 mL iPrOH, and 20 μL 5% Nafion 117 solution (Sigma Aldrich). The electrode layer was deposited by dropping 10 μL increments of the catalyst suspension onto the FTO substrate until a total catalyst loading of 100 μg cm^−2^ was reached. The deposited catalyst layer was dried in between each drop-casting step. The electrochemical experiments were performed in a three-electrode setup using the respective substituted TiO_2_/FTO working electrode in combination with a platinum mesh as the counter electrode and a saturated calomel electrode (SCE) as the reference electrode. For potential control, an AUTOLAB (PGSTAT 30) potentiostat was used in all experiments. Voltammograms were recorded at a polarization rate of 5 mV s^−1^ in 0.1 M HClO_4_ in a potential range of 1.3 to 2.0 V *vs.* RHE. Linear polarization curves were measured after collecting CVs at a scan rate of 50 mV s^−1^ to achieve a constant double layer charge.

All potentials were recalculated and reported in the reversible hydrogen electrode (RHE) scale. Electrochemical impedance spectroscopy measurements were recorded in the range from 15 kHz to 1 Hz with an amplitude of 10 mV to estimate the ohmic drop of the solution. All voltammograms were corrected for uncompensated solution resistance. The reported current densities are based on the geometric surface area of the electrodes used. All electrochemical measurements were repeated at least three times to ensure reproducibility. DEMS experiments were carried out in a home-made Kel-F single compartment cell in a three-electrode set-up using a Pt-auxiliary and an Ag/AgCl reference electrode.^68^ All DEMS experiments were controlled using a PAR 263A potentiostat. The DEMS experimental setup comprises a PrismaPlusTM QMQ220 quadrupole mass spectrometer (Pfeiffer) connected to a HiPace 80 (PMP03941) turbomolecular drag pumping station (Pfeiffer). ICP-OES measurements were performed using an Agilent 5100 dual-view instrument fitted with a Seaspray nebulizer, double-pass cyclonic spray chamber, and 2.8 mm wide bore torch for high total dissolved solids applications, using a flow-through electrochemical cell of our own design as detailed elsewhere.^59^

## Conclusions

Simultaneous substitution of wide bandgap semiconductors with both n- and p-type dopants affects both the electronic structure as well as the catalytic behavior of semiconducting oxides. This co-substitution significantly improves the OER activity of these materials. TiO_2_ and SrTiO_3_, known for their stability under acidic and alkaline conditions and their poor performance in OER catalysis, were chosen as model systems to outline the co-substitution effect computationally. The inclusion of both n-type and p-type dopants in these structures causes a compensation effect between the electronic states of the dopants, resulting in an alteration of the band structure, which consequently lowers the overpotential of OER. According to the pDOS, the dopants form a new band that aligns the Fermi level at an energetic position favorable for water oxidation. The calculations further reveal a sensitivity of the binding energies of the intermediates to the relative position of the dopants. Combining this local structure sensitivity with the Sabatier principle can be used to optimize the chemical composition of the co-substituted catalysts. The theoretical predictions were confirmed experimentally on the substituted and co-substituted TiO_2_ – anatase. The formation of the pseudo-conductor band is reflected in the significant coloration of the substituted anatase. While a similar change in the electronic structure can be seen for a single-substituted system, only n- and p-type co-substituted materials show an actual significant improvement of the catalytic activity in oxygen-evolving reactions.

## Data availability

Theoretical structures and scripts necessary for reproducing the results obtained herein, as well as experimental data, have been made freely accessible at KatlaDB – Theoretical Catalysis Database – and can be accessed *via*https://nano.ku.dk/english/research/theoretical-electrocatalysis/katladb/codoping-TiO_2_.

## Author contributions

CRediT (contributors roles taxonomy) was used for standardized author contributor role description. T. K., S. D.: data curation, formal analysis, investigation, methodology, software, visualization, writing – original draft. R. P.: data curation, formal analysis, investigation, methodology, visualization, validation, supervision, writing – original draft, writing – review & editing. R. M., A. M. F., K. M.-M., R. N.; D. Z.: data curation, formal analysis, visualization, writing – review & editing. S. F. L. M., H. H.: resources, supervision, methodology, funding acquisition, writing – review & editing. P. K., J. R.: project administration, conceptualization, resources, supervision, methodology, funding acquisition, writing – review & editing.

## Conflicts of interest

There are no conflicts to declare.

## Supplementary Material

SC-013-D2SC04585K-s001
