# Supplementary material for: Synergistic effect of p-type and n-type dopants in semiconductors for efficient electrocatalytic water splitting
Source: Chem Sci. 2022 Nov 15;13(46):13879–92. doi: 10.1039/d2sc04585k (PMC9710220; doi:10.1039/d2sc04585k)
Supplement: SC-013-D2SC04585K-s001 [file SC-013-D2SC04585K-s001.pdf]

Electronic Supplementary Material (ESI) for Chemical Science.
This journal is © The Royal Society of Chemistry 2022

SUPPORTING INFORMATION

Synergistic effect of p-type and n-type dopants in semiconductors for efficient electrocatalytic water splitting

Tugce Kutlusoy[‡a], Spyridon Divanis[‡a], Rebecca Pittkowski[a,b], Riccardo Marina[c], Adrian M. Frandsen[a], Katerina Minhova-Macounova[b], Roman Nebel[b], Dongni Zhao[d], Stijn F. L. Mertens[d], Harry Hoster[d,e], Petr Krtil[b] and Jan Rossmeisl[*a]

a Center of High Entropy Alloy Catalysis, Department of Chemistry, University of Copenhagen, Universitetsparken 5, 2100 København Ø, Copenhagen, Denmark.

b J. Heyrovsky Institute of Physical Chemistry, Academy of Sciences of the Czech Republic, Dolejskova 3, Prague 18223, Czech Republic

c New Application Research, Research and Development Division, Industrie De Nora S.p.A., 20134 Milan, Italy

d Department of Chemistry, Energy Lancaster and Materials Science Institute, Lancaster University, Lancaster LA1 4YB, United Kingdom

e Fakultät für Ingenieurwissenschaften, Lehrstuhl Energietechnik, Universität Duisburg-Essen, Lotharstra. 1, 47048 Duisburg, Germany

‡ Both authors contributed equally to this manuscript

* Corresponding author

Coulombic effect

The Coulombic effect can be shown on the OER volcano: By varying the position of dopants in the structure, the theoretical overpotential can be modified over a wide range of potentials. The position of structures on the OER activity volcano directly depends on the distance of the dopants from the intermediate. ***Fig. S1*** demonstrates this on the example of TiO_2, where the structure was co-doped with Rh and V by placing two dopants of each substituting element and randomly varying their respective position within the TiO_2 slab. The Coulombic effect is also clearly observed when only n-type dopants are used as dopants in TiO_2 and their position is varied within the structure [see ***Fig. S3*** for V, ***Fig. S5*** for Ta, ***Fig. S6*** for Nb, and ***Fig. S7*** for W

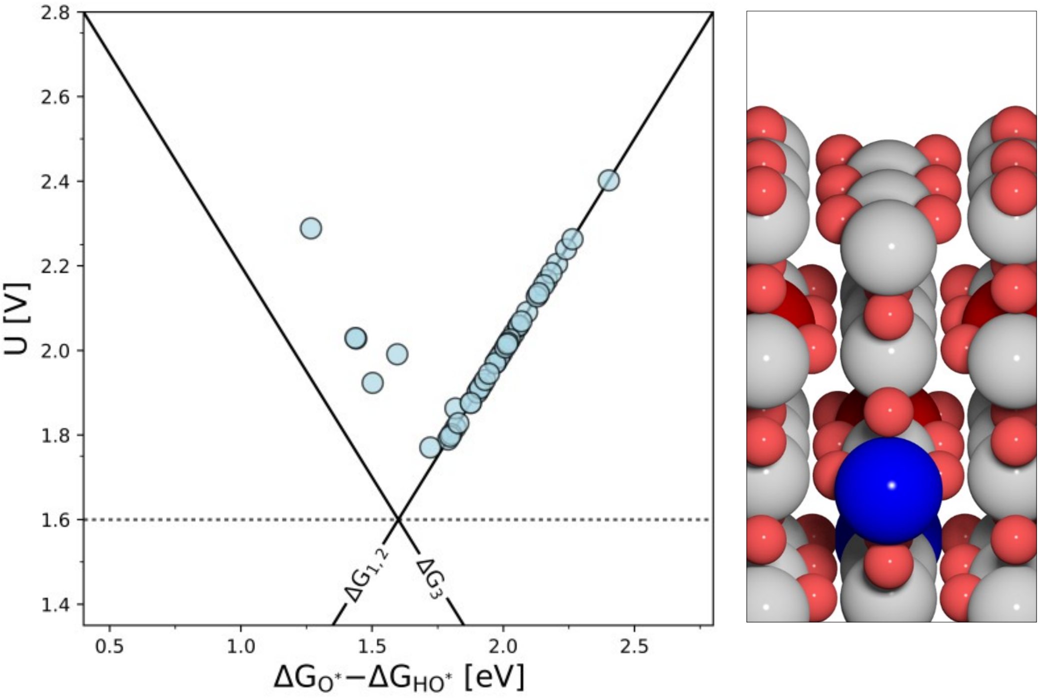

and Mo]

Fig S1. *OER volcano (right) showing the theoretical OER activity of various Rh-V co-doped TiO_2 structures with random placements of the dopants (two V and two Rh) within the TiO_2 structure. One of the random configurations generated is shown on the left, V is displayed in red, Rh is displayed in blue.*

a)

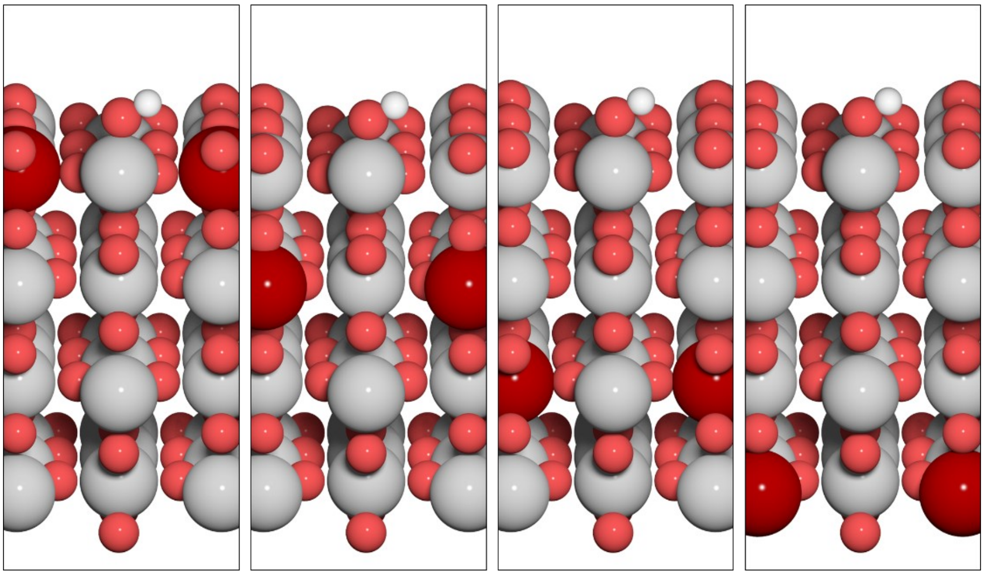

b)

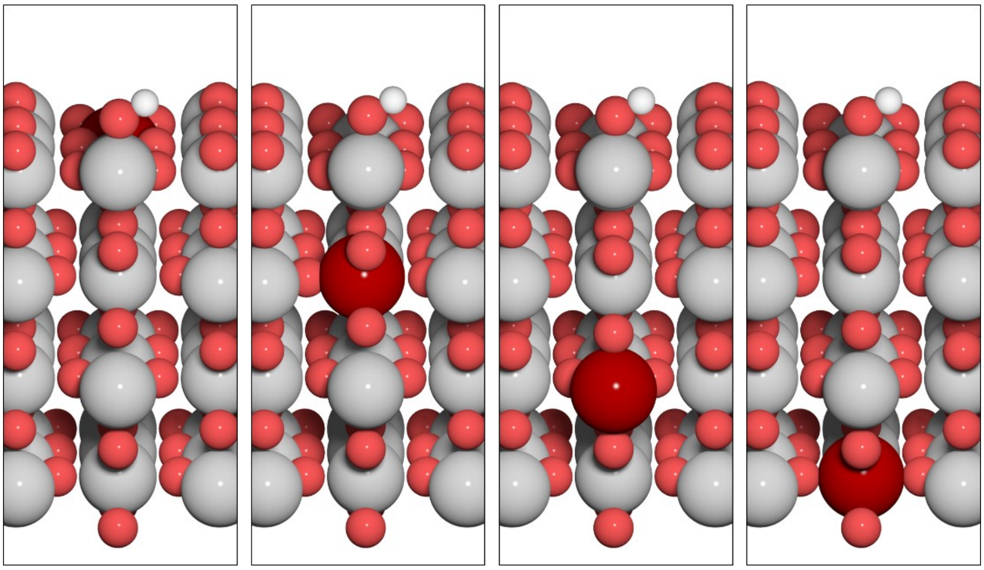

Fig S2. *TiO_2 structures including one V dopant in* ***a)*** *the bridge sites and* ***b)*** *the cus site (of the surface) which is continuously moved through the layers of the slab to vary the distance between the dopant and the OH adsorbate.*

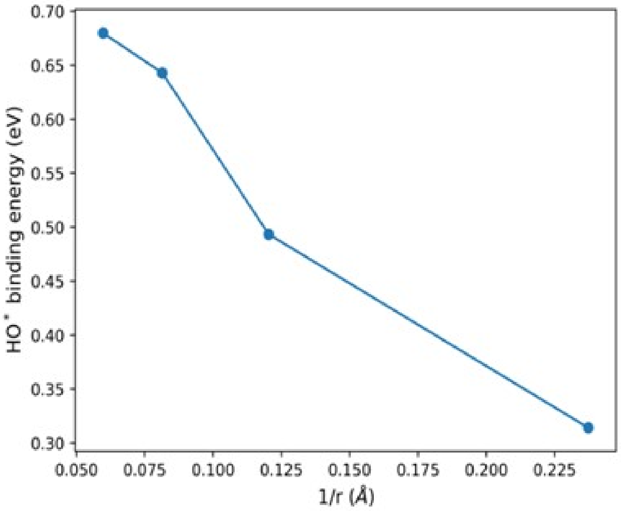

Fig S3. *Schematic representation of the effect of the distance between the intermediate (HO*) and the dopant (V) on the binding energy of the intermediate in a $SrTiO_3$ structure.*

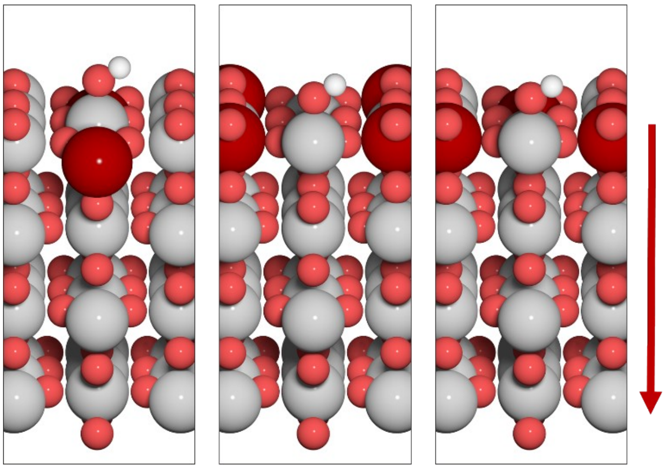

Fig S4. *Structural representation of rutile TiO_2 (110) doped with two dopants, where the position of the dopants is varied throughout the slab to estimate the adsorbate binding energy (here OH) as a function of distance to the dopant. The two dopants are either placed both in the cus-position (left), placed both in the bridge position (middle), or one dopant placed in cus and one in bridge position of the surface. While they are moved away from the surface through the bulk the position names are kept as cus, bridge, and cus+bridge, respectively.*

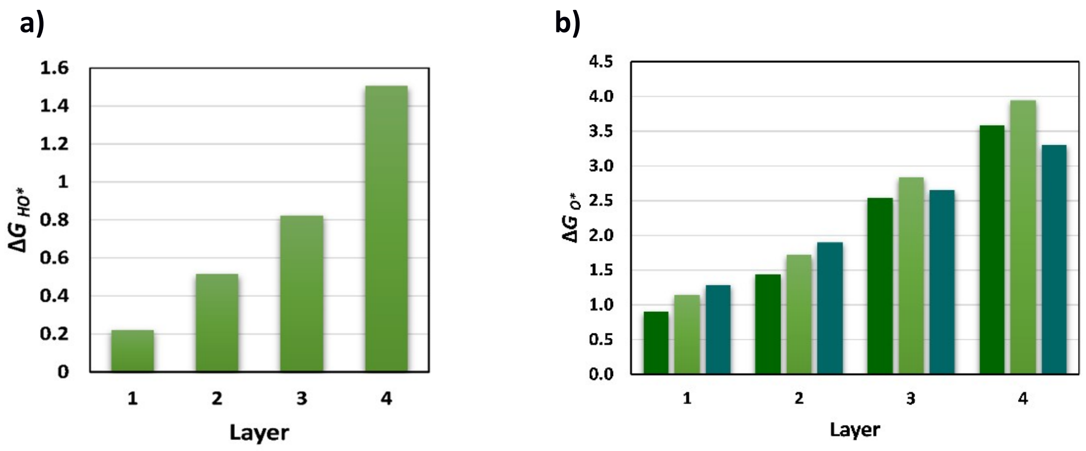

Fig S5 **a)** *The HO* binding energy against the placement of one V dopant at different sublayers in the* TiO_2 *structure* ***b)*** *The O* binding energy placing two V dopants at different* TiO_2 *sublayers with substitution at the cus (left), cus+bridge (middle) and bridge (right) sites in the* TiO_2 *structure.*

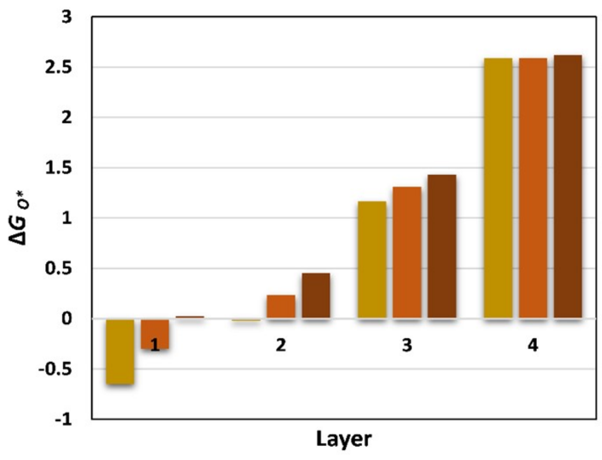

Fig. S6 The O* binding energy for placing two Ta dopants at different TiO_2 sublayers with substitution at the cus (left), cus+ bridge (middle), and bridge (right) sites in the TiO_2 structure.

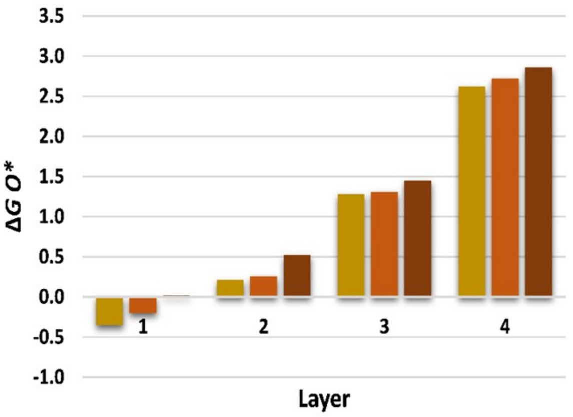

Fig. S7 The O* binding energy for placing two Nb dopants at different TiO_2 sublayers with substitution at the cus (left), cus+ bridge (middle), and bridge (right) sites in the TiO_2 structure.

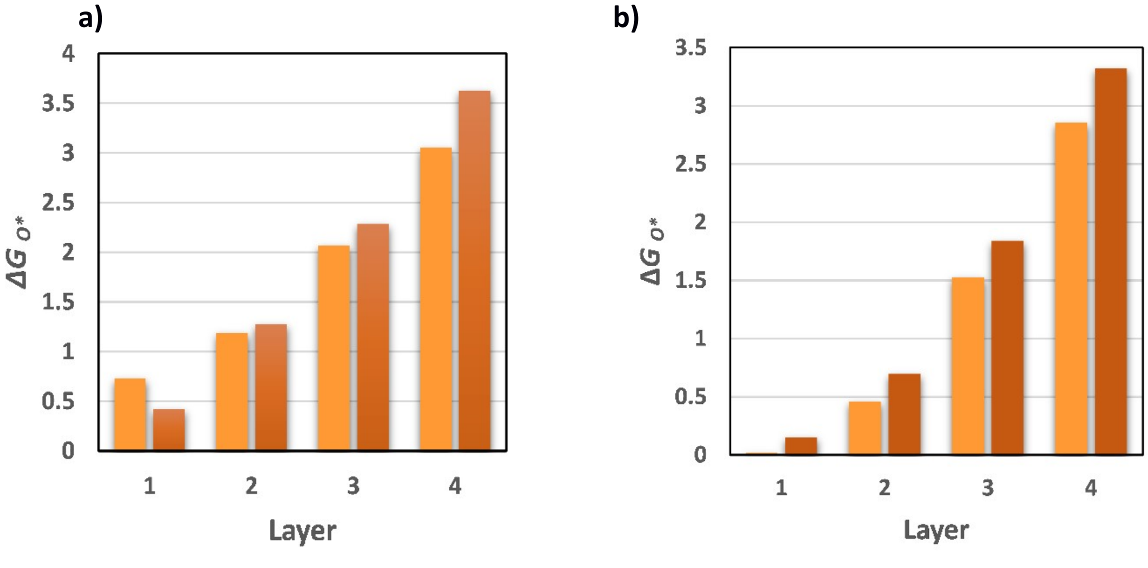

Fig. S8 O* binding for placing a) one Mo dopant and b) one W dopant in different layers of TiO_2 at the cus (left) and bridge (left) sites, respectively.

The Coulombic effect is, however, less pronounced when only p-type dopants are incorporated into a structure. As a consequence of inserting the p-type dopant, the Fermi level shifts to the valance band of the structure. Thus the electrons contributing

to the chemical bonds between the surface and the intermediates have almost the same energy, hence not a large variety of binding energies is observed as seen in the example of Rh-substitution in ***Fig. S9***. Further examples for Pd, Mn, and Ru-substitution are shown in ***Fig. S10***.

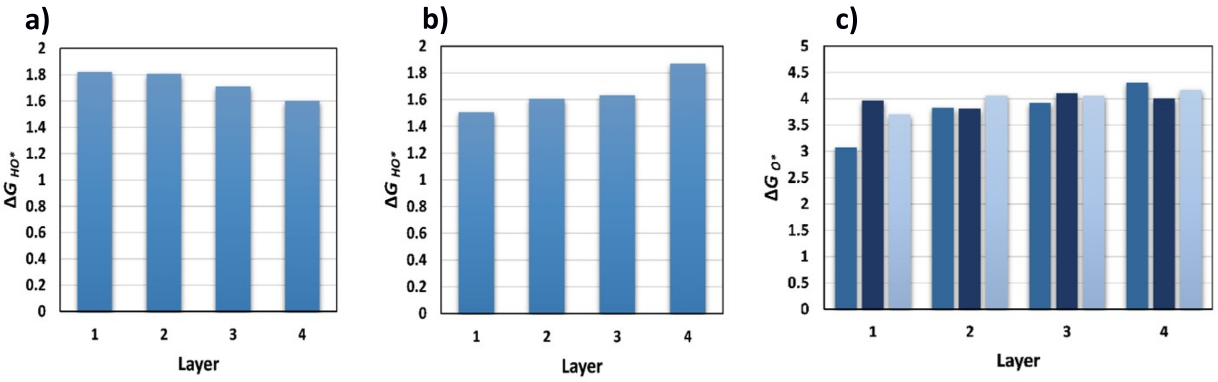

Fig. S9 *HO* binding energy for positioning one Rh dopant in different layers of TiO_2 in a) the cus site and b) the bridge sites. c) The O* binding energy for placing two Nb dopants at different TiO_2 sublayers with substitution at the bridge (left), cus (middle), and cus+bridge (right) sites in the TiO_2 structure.*

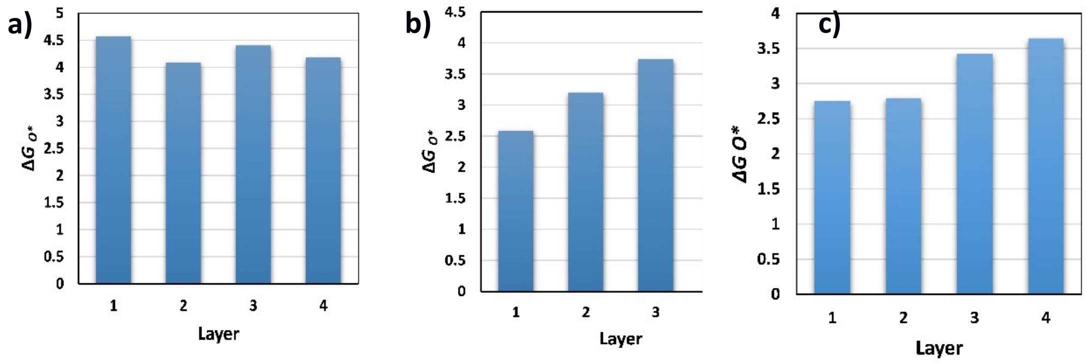

Fig. S10 *O* binding for positioning a) two Pd, b) two Mn, and c) two Ru dopants in the cus sites of different layers of TiO_2.*

a) b)

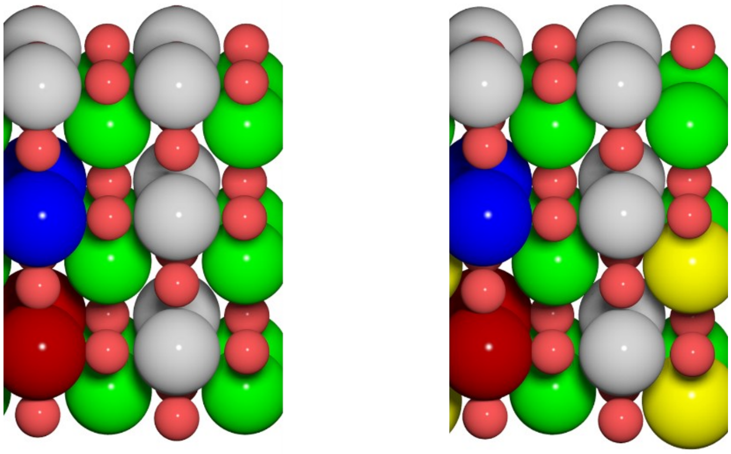

Fig. S11 *Structural representation in (100) orientation of the V-Rh co-doped cubic perovskites* ***a)*** *$SrTiO_3$ and* ***b)*** *$Ba_{0.16}Sr_{0.84}TiO_3$ with the color scheme Sr in green, V in red, Rh in blue, and Ba in yellow.*

Extra substituent pairs

The scaling relation that is found between HO* and HOO* is equal to 3.58 eV instead of 3.2 eV. This has previously been reported [S1] and has been attributed to the electronic nature of TiO_2. The different structures are placed both on the strong and on the weak binding side of the volcano, including at the top of the new volcano which is constructed from the different HO*/ HOO* scaling relation on TiO_2.

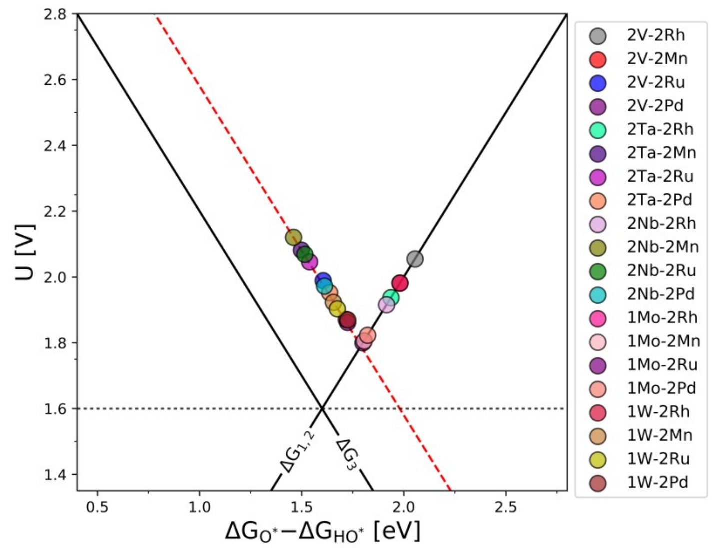

Fig S12. *Different couples of p- and n-type dopants in a TiO_2 structure. The dashed line corresponds to the energy interdependency of the HO* and HOO* intermediates.*

U-correction effect on the binding energies of the intermediates

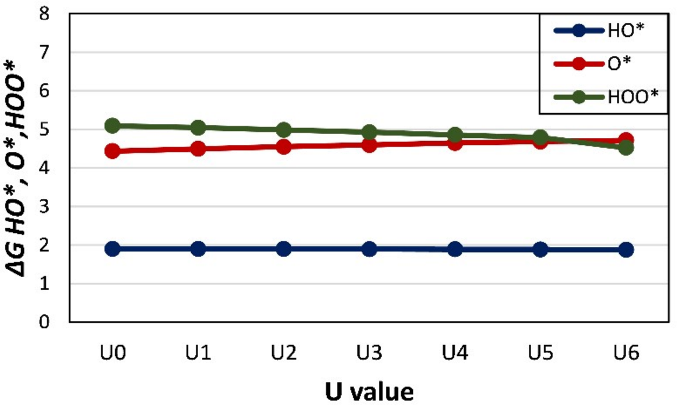

Fig. S13 *Adsorption energy of OER intermediates (HO*, O*, HOO*) on pristine TiO_2 as a function of varied U-values starting from 0 to 6.*

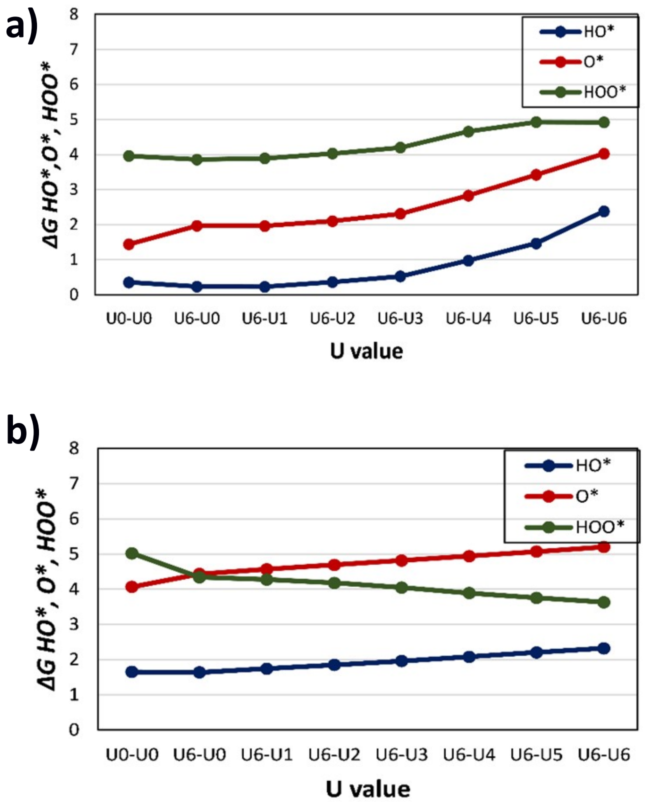

Fig. S14 a*)* *Adsorption energy of OER intermediates (HO*, O*, HOO*) on vanadium-doped* TiO_2 *as a function of varied U-values for V starting from 0 to 6, the U-value for* TiO_2 *is fixed to U=6.* ***B)*** *Adsorption energy of OER intermediates (HO*, O*, HOO*) on rhodium-doped* TiO_2 *as a function of varied U-values for V starting from 0 to 6, the U-value for* TiO_2 *is fixed to U=6.*

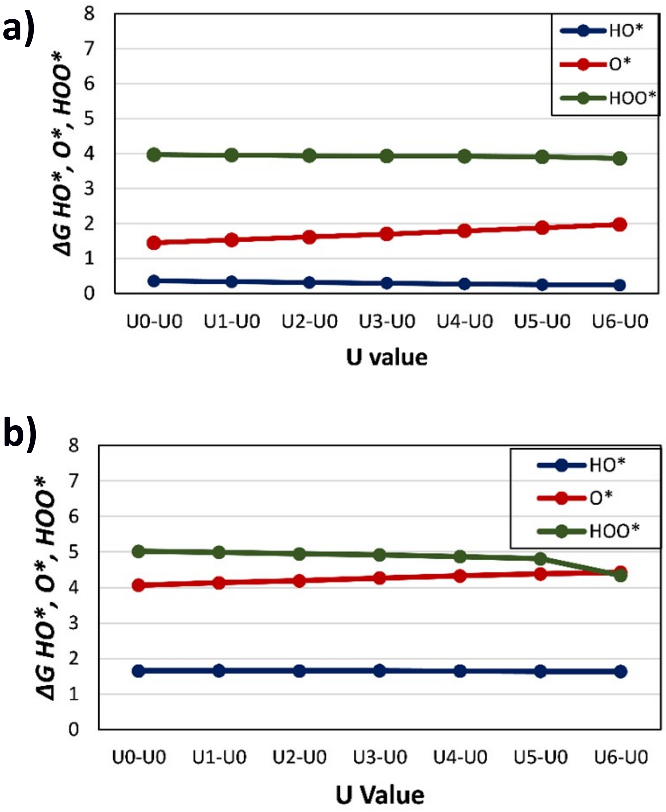

Fig. S15 a) *Adsorption energy of OER intermediates (HO*, O*, HOO*) on vanadium-doped* TiO_2 *as a function of varied U-values for* TiO_2 *starting from 0 to 6, the U-value for the V-dopant is fixed to U=0.* ***b)*** *Adsorption energy of OER intermediates (HO*, O*, HOO*) on rhodium-doped* TiO_2 *as a function of varied U-values for* TiO_2 *starting from 0 to 6, the U-value for the Rh-dopant is fixed to U=0.*

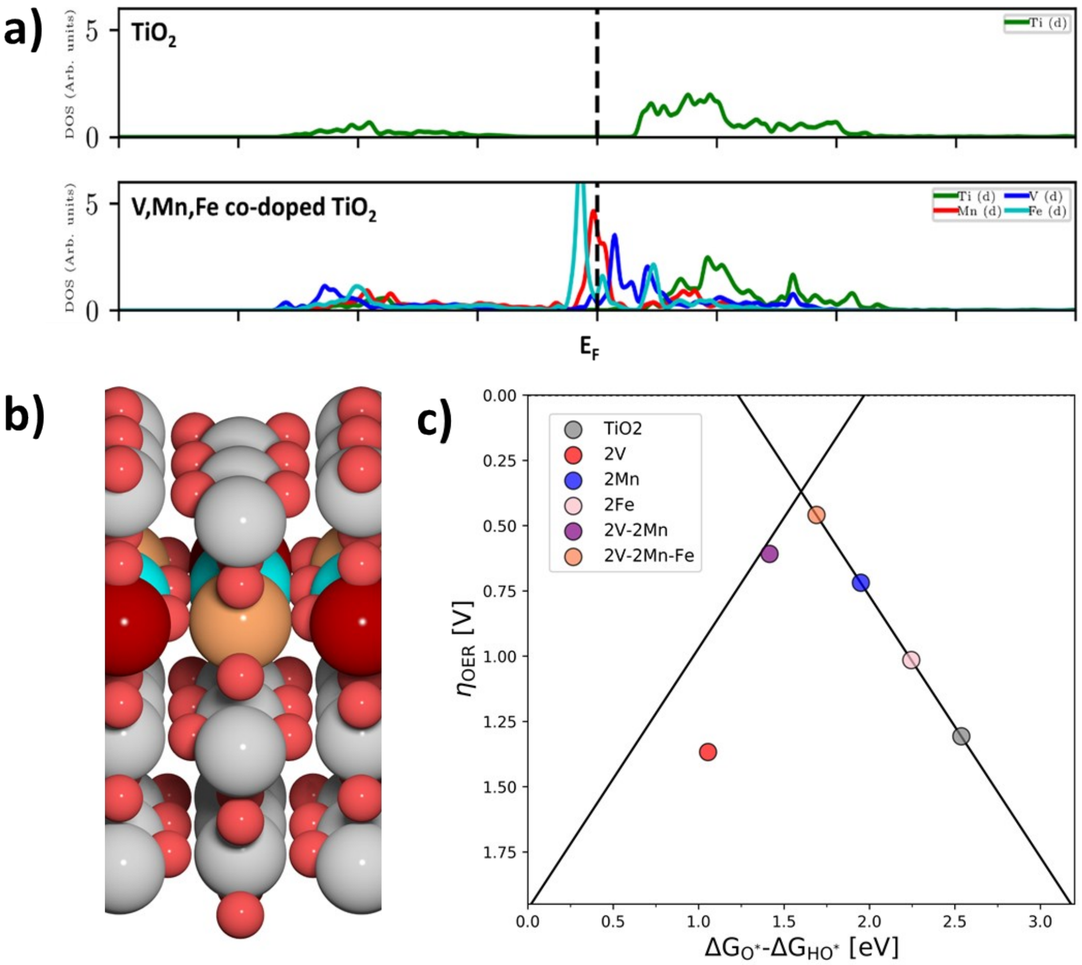

Fig. S16 a) *Projected density of states of pristine and Mn, V, and Fe co-doped TiO_2. The additional d-states of the dopants are mainly located at the Fermi level* ***b)*** *geometry optimized structure of TiO_2 slab with Mn (cyan), V (red), and Fe(orange)-dopants* ***c)*** *theoretical OER activity volcano showing the decrease in theoretical overpotential through introducing additional dopants into the do-doped structure***.**

Co-substitution on anatase polymorph

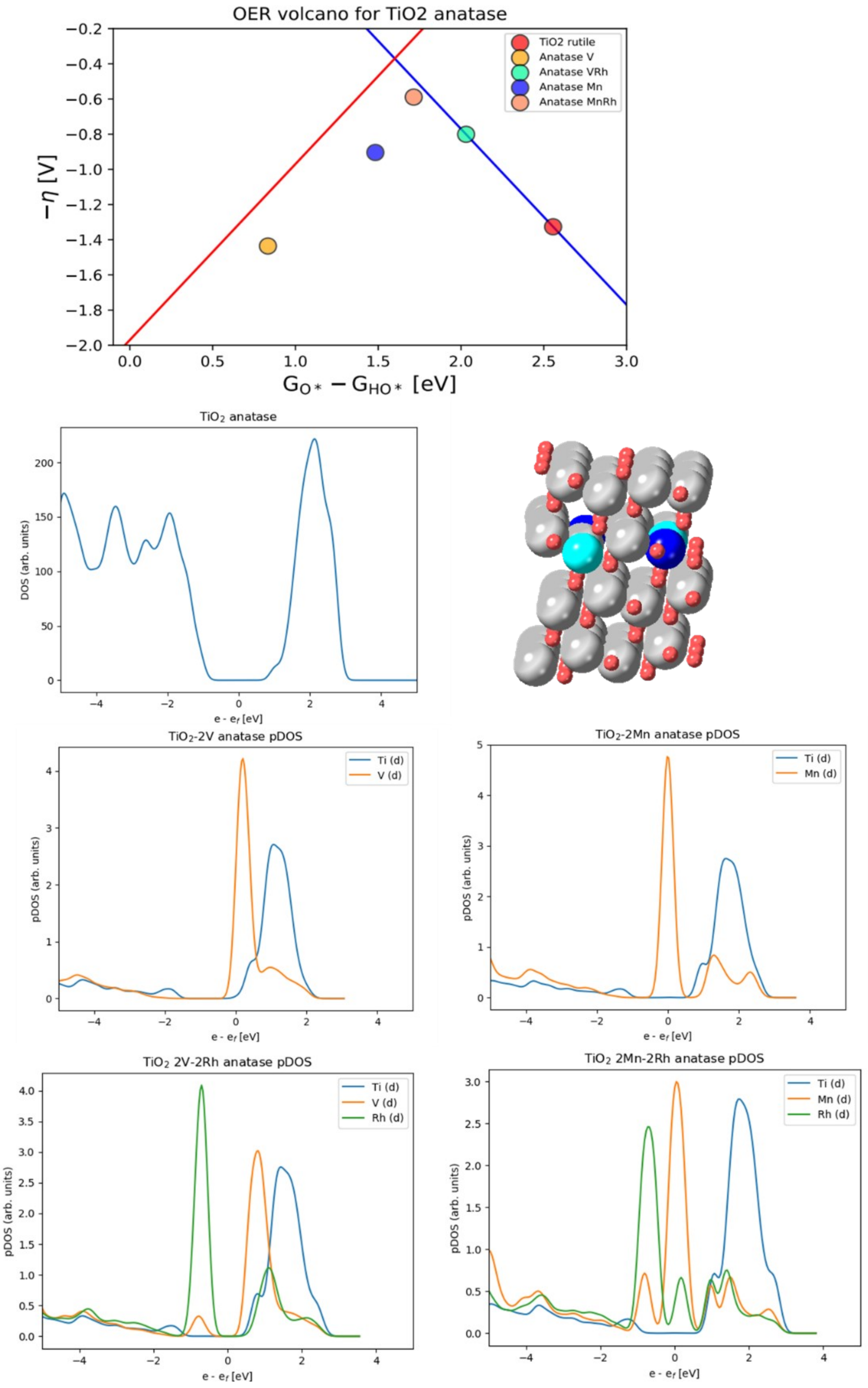

Fig. S17 *Theoretical OER activity volcano showing the decrease in theoretical overpotential through introducing additional dopants into the co-substituted anatase structure, and projected density of states of pristine and Mn, V substituted anatase TiO_2 as well as Mn/V and Mn/Rh co-substituted anatase TiO_2, and geometry optimized structure of TiO_2 anatase slab substituted with Mn (cyan), and Rh (blue).*

Structural Characterization

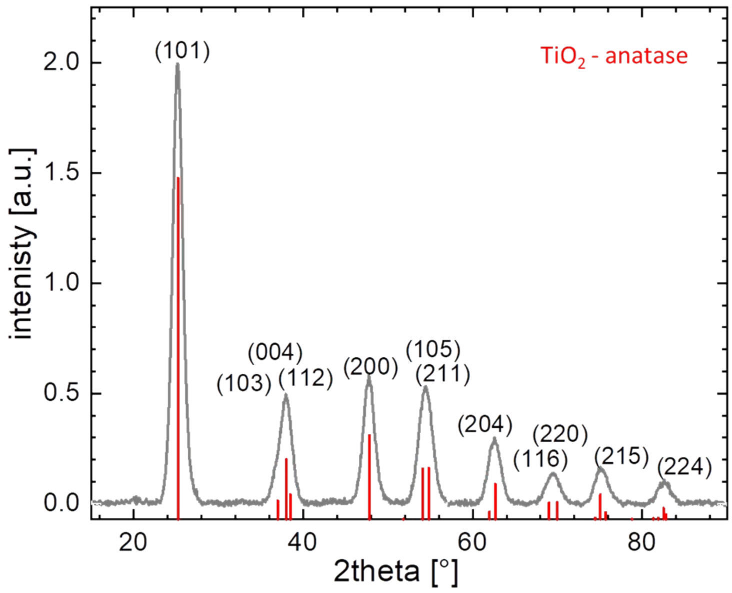

Fig. S18 *Typical diffraction pattern of a co-doped TiO_2 sample (V/Cr co-doped, 20 at.% of dopants) indexed with the reflections of TiO_2 in the anatase polymorph.*

The diffraction pattern (see **Fig. S18**) shows that only one crystalline phase is obtained in the synthesis, which can be assigned to the tetragonal anatase structure ($I4_1$/amd, 141). Due to the pure crystallinity of the nanocrystals obtained, the diffraction patterns measured are of poor quality and do not show the typical peak splitting expected for anatase structure. For example, the peak in the diffraction pattern located at 38 °2theta is typically a triplet of the (103), (004), and (112) reflections. Slight variations in the lattice parameters *a* and *b* are evident from the Rietveld refinement, as is expected for doped TiO_2 structures (see **Table S1**). The Scherrer coherent domain size was estimated for all prepared materials and ranges between 8 and 13 nanometers (see **Tables S1**). The Mn/Co substituted sample shows a large, asymmetric shoulder around 35 °2theta on the Bragg peak of the (103), (004), and (112) triplet. This could indicate presence of a MnOx or CoOx side phase, as several of the cobalt and manganese oxides also have Bragg peaks at similar diffraction angles (see Fig S18). Rietveld refinement of the diffraction data yields however no phase contribution from any of the cobalt or manganese oxides, as the peak can be described by contribution from the broadened (103) Bragg peak and no other Bragg peaks that could be attributed to the manganese or cobalt oxides is present in the diffraction pattern (see **Fig. S19**). It can however not be excluded that a minor contribution of another oxide is present in the sample.

Typical SEM micrographs are depicted in **Fig S20**, which confirm nanoparticles of around 10 nm average size. The concentration of substituents in the synthesized structures was determined by Energy-dispersive X-ray spectroscopy (EDX). The EDX analysis confirms that the actual amounts of dopants in the structure agrees well with the nominal composition of the precursor solutions (see **Table S1**). After electrolysis, the used electrodes lose a small fraction of the substituents, but the substituent concentration is largely retained, which agrees with the stability analysis as followed by ICP-OES.

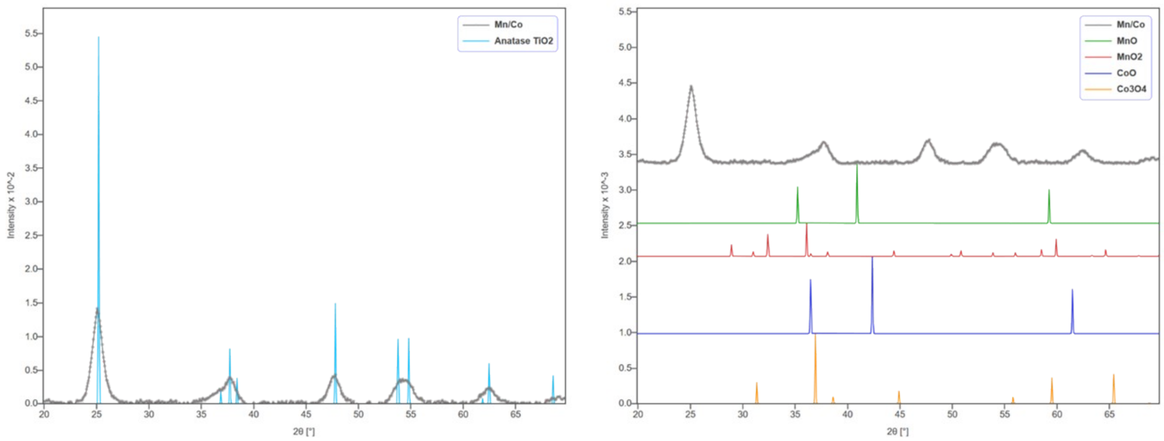

Fig. S19 *Diffraction pattern of the Mn/Co substituted anatase structure, which exhibits a shoulder around 35 °2theta, which could indicate the occurrence of other oxides in minor amounts. While both manganese and cobalt oxides scatter at similar angles, other characteristic Bragg peaks that would confirm their presence are, however, absent.*

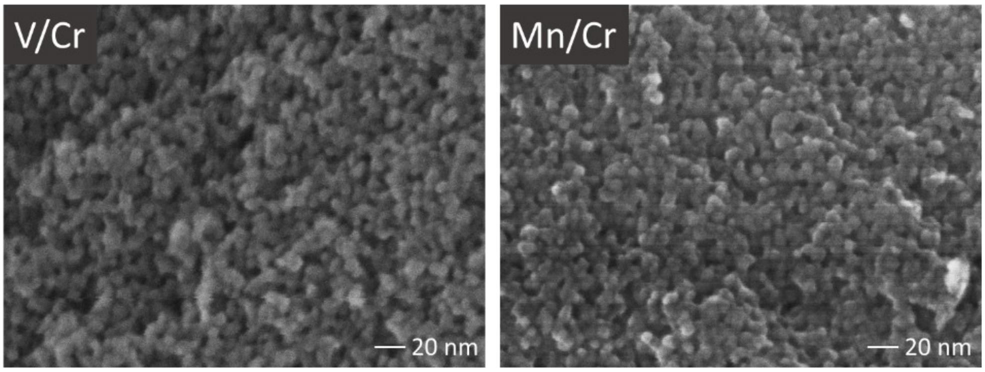

Fig. S20 *Typical SEM micrographs of the synthesized co-doped TiO_2 nanoparticles showing the V/Cr and Mn/Cr co-doped samples.*

Table S1 *Details of the structural characterization of obtained doped TiO_2 nanoparticles: unit cell parameters a and b as obtained from the Le Bail fits of the X-ray diffraction patterns and the figures of merit of the refinement (R_{wp}, R_{exp}, χ^2, and GOF), domain size of the nanoparticles as determined from the XRD patterns using the Scherrer equation, and amount of respective dopants in the sample as determined by EDX analysis of the initial powders and of the used electrodes (marked in brackets).*

	a **[Å]**	***b*** **[Å]**	R_{wp}	R_{exp}	χ^2	**GOF**	**Domain Size [nm]**	**Dopant amount (EDX) [at%] of powder & (used electrode)**
**Mn/V**	3.801(1)	9.526(3)	5.32	3.94	1.82	1.35	8	11.2±0.6 V, 9.0±0.3 Mn<br>(9.5 ±0.9 V, 7.5±0.9 Mn)
**V/Cr**	3.803(1)	9.483(4)	8.21	3.87	4.50	2.12	8	11.0±1.3 V, 8.7±1.6 Cr<br>(10.0±1.1 V, 9.4±1.2 Cr)
**Mn/Co**	3.799(2)	9.509(4)	2.69	2.42	1.23	1.11	9	9.0±0.4 Mn, 10.1±0.5 Co<br>(9.6±1.0 Mn, 12.2±1.6 Co)
**Mn/Fe**	3.804(1)	9.521(1)	3.82	3.46	1.22	1.10	11	10.2±0.4 Mn, 8.1±0.5 Fe<br>(10.0±1.1 Mn, 8.5±0.7 Fe)
**Mn**	3.800(2)	9.505(6)	4.47	3.77	1.41	1.18	10	19.6±0.8 Mn<br>(22.0±3 Mn)
**Cr**	3.803(1)	9.484(2)	5.23	3.80	1.89	1.37	8	17.9± 1.6 Cr<br>(20.1±4 Cr)

Fig. S21 Elemental Maps from obtained from SEM/EDX mapping of the TiO2 samples substituted Mn and V with as well as the sample substitutes with Mn and Co.

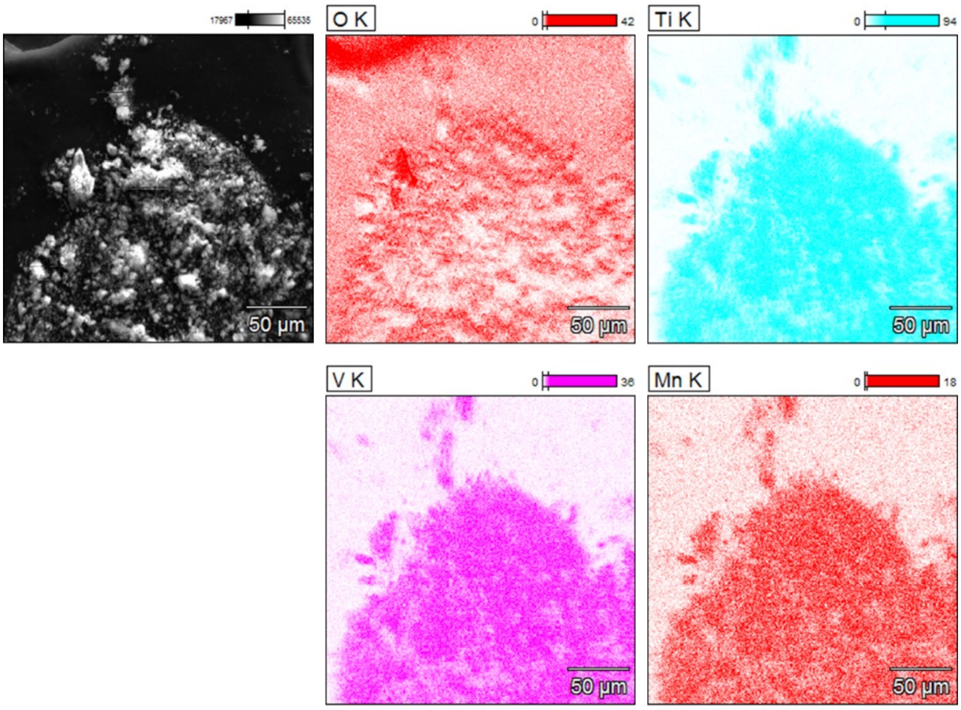

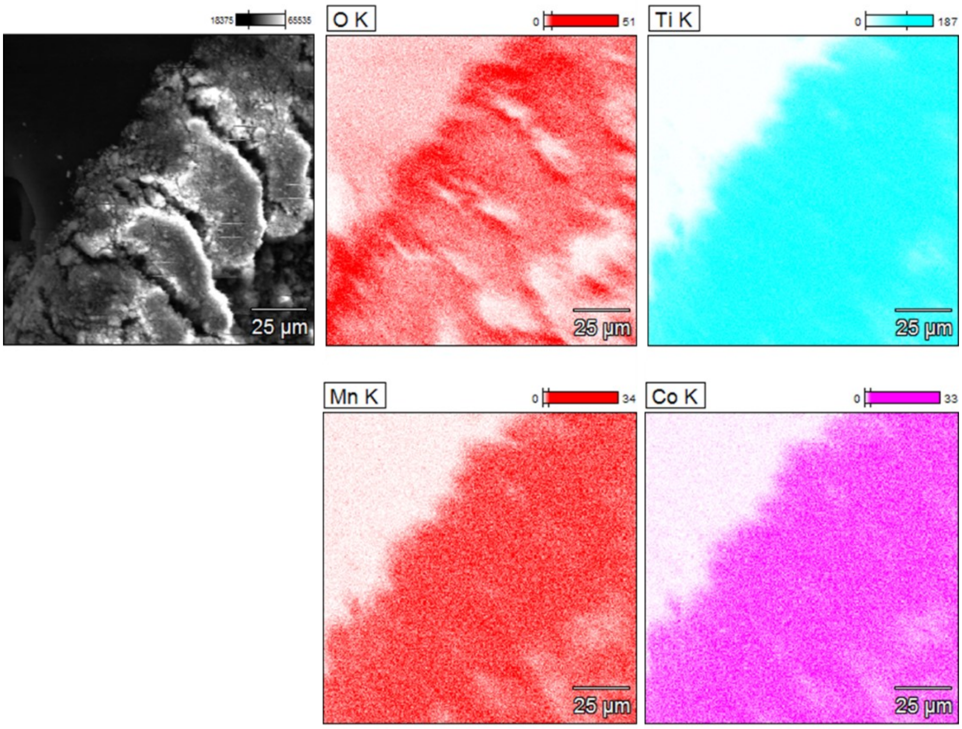

References:

[S1] Huang, X.; Wang, J.; Tao, B.H.; Tiana, H.; Xu, H. An essential descriptor for the oxygen evolution, Chem. Sci., 2019, 10, 3340.
